# Supplementary material for: Evaluating the efficacy of various traps in catching tsetse flies at Nech Sar and Maze National Parks, Southwestern Ethiopia: An Implication for Trypanosoma Vector Control
Source: PLoS Negl Trop Dis. 2022 Dec 22;16(12):e0010999. doi: 10.1371/journal.pntd.0010999 (PMC9822101; doi:10.1371/journal.pntd.0010999)
Supplement: Attachment — Submitted filename: Corrected_Line by line response to reviewers_1.docx [file pntd.0010999.s001.docx]

**Line by line response to reviewers’ questions and comments**

**Methods**

Reviewer #1: The objectives are clearly articulated and the design (Latin Square) is appropriate for assessment of field responses of tsetse the traps. I am however concerned about the statistical approach employed in analysis of the data.  Given that the data was discrete/count of flies (non-continuous), ANOVA is not the appropriate tool for the analysis, unless the data was transformed. Non-parametric methods would have been most appropriate.

**Response:** Thank you for noticing this problem. We checked the data normality, it was much skewed, so we used the nonparametric test (Kruskal-Wasllis Test pairwise comparison and Mann-Whitney U Test) to analyse the data rather than ANOVA test.

Reviewer #2: -The objectives of the study are clearly articulated and the study design is appropriate
-The statistical analyses used were not appropriate for the type of data collected. Tsetse catches are counts and thus cannot be analyzed using General Linear Models like ANOVA (which are suitable for continuous variables) without being transformed. Generalized Linear Models are more suitable for counts.

**Response:** Thank you for noticing this problem. We checked the data normality, it was much skewed, so we used the nonparametric test (Kruskal-Wasllis Test pairwise comparison and Mann-Whitney U Test) to analyse the data rather than the generalized linear model like ANOVA.

Reviewer #3: The paper is clear and very well written, and gives insight into the efficiency of different types of traps in catching tsetse flies at different areas and vegetation types. This evaluation is important because will allow to determine the real impact of control programs implemented in the region. The materials and methods are clearly indicated.

**Results**
-Does the analysis presented match the analysis plan?
-Are the results clearly and completely presented?
-Are the figures (Tables, Images) of sufficient quality for clarity?

Reviewer #1: The results are well written and presented.

Reviewer #2: Due to the flawed statistical analyses indicated above, the results are unreliable.
**Response**: Thank you again. The data normality was checked, it was much skewed, so we used the nonparametric test (Kruskal-Wasllis Test pairwise comparison and Mann-Whitney U Test) to analyse the data rather than ANOVA test.

Reviewer #3: The results reflect to the methodology are clearly presented.

**Conclusions**
-Are the conclusions supported by the data presented?
-Are the limitations of analysis clearly described?
-Do the authors discuss how these data can be helpful to advance our understanding of the topic under study?
-Is public health relevance addressed?

Reviewer #1: The conclusions are appropriate and are supported by the data presented. However, the data might need to be analysed again using appropriate tool.

**Response:** the appropriate statistical tool is employed based on the previous comments.

Reviewer #2: The conclusions could be erroneous as the results are unreliable due to the flawed statistical analysis

**Response:** We used the nonparametric test rather than ANOVA

Reviewer #3: The results are also adequately discussed.

**Editorial and Data Presentation Modifications?**
Use  this section for editorial suggestions as well as relatively minor modifications of existing data that would enhance clarity. If the only modifications needed are minor and/or editorial, you may wish to recommend “Minor Revision” or “Accept”.
Reviewer #1: The results are well written and presented.
Reviewer #2: (No Response)
Reviewer #3: Minor revision

**Summary and General Comments**
Use  this section to provide overall comments, discuss strengths/weaknesses of the study, novelty, significance, general execution and scholarship. You may also include additional comments for the author, including concerns about dual publication, research ethics, or publication ethics. If requesting major revision, please articulate the new experiments that are needed.

Reviewer #1: The MS by Asfaw et al., present a very clear and straightforward comparative assessment of efficacy of the three different traps (Ngu, Biconical and sticky) in four ecological zones (wood grassland, bush land, forest, and riverine forest) using tried, tested and approved methods.  The findings are well written and presented. I am, however, concerned about two major issues. First, the statistical tool used for analysis of the data (ANOVA) was inappropriately applied or should have been substituted by a non-parametric method suitable for the count data that was analysed. The second concern is the lack of novelty in the findings, given that the results only appears to confirm other results, what is already known about relative performance of these devices as captured by the authors in the discussions and elsewhere. G. pallidipes is a savannah tsetse species while G. fuscipes is riverine species, information which is well documented, and which the authors appear to
confirm.  Other minor concerns is that the " personal communication" in line 254 should be qualified.

**Response:** The statistical method for analysis is corrected as to the previous comments. The novelty of this work is to show the efficiency of those traps during the last miles of tsetse control interventions in various vegetations. This conveys the most important message to improve the economical and interventional efficiency of control programs using the appropriate trap types. The “personal communication” in line 254 is also removed to avoid confusions.

Reviewer #2: The manuscript requires substantial improvement in grammar and the authors should consult a statistician for their data analysis.

**Response:** Some grammatical corrections are made accordingly. The statistically appropriate method is also employed through the support of a statistician.

Reviewer #3: This is a technical report about “Evaluating the Efficacy of Various Traps in Catching Tsetse Flies at Nech Sar and Maze National Parks, South-western Ethiopia : An Implication for  Trypanosoma Vector Control”.

The paper is clear and very well written, and gives insight into the efficiency of different types of traps in catching tsetse flies at different areas and vegetation types. This evaluation is important because will allow to determine the real impact of control programs implemented in the region. The materials and methods are clearly indicated and the results reflect this. The results are also adequately discussed.
It is recommended that the manuscript be accepted for publication.

Some minor errors and modifications are suggested here below.

Abstract
Line 34: remove “s” to traps in “…sticky traps”

**Response**: removed
Author summary
Line 46: add “African” before “Animal trypanosomosis”

**Response**: Added
Line 46: add “agents” after “Animal trypanosomosis (Nagana) and Human African Trypanosomiasis (HAT)…”

**Response**: Modified
Line 47: add “In animals,” before “…The disease is arguably the main constraint, …”

**Response**: modified this way…. African Animal Trypanosomiasis…
Line 50: “to control the disease" which disease (HAT or AAT)? Please precise

**Response**: Modified as both HAT and AAT

Introduction
Line 76: remove “s” at the end of “SITs”

**Response**: removed

Line 86: put “,” after “In Ethiopia”
Materials and methods
Line 109: change “0” by “°”    “…200C and 340C….”

**Response**: Changed
Figure 1: Kulfo and Maze rivers must be displayed along their lengths on the map

**Response:** full improvements on Maze river is made. Kulfo river is improved to its maximum length because it sources out from a mountain and feeds Lake Abaya (Abaya Hayk) at the end.

Line 126-127: the 12 traps were deployed in each study site or each vegetation? Please clarify the sentence.

**Response**: Corrected as each vegetation

Lines 127-128: change “In the six consecutive months, a total of 72 traps per study site were deployed.” to “In the six consecutive months, a total of 72 traps were deployed per study site.”

**Response**: Corrected as stated

Lines 129-130: the sentences “The type of trap used at the first trapping point in each type of vegetation was chosen at random.” has been repeated, please delete.

**Response**: Deleted
Line 137: for six months collection, the authors indicated that this makes possible to observe the seasonal variation in fly density.

**Response**: Season deleted, only monthly fly density
I am not sure that this is enough to observe the seasonal variation. This will be possible if the experiments were conducted over the 12 months.
Data management and analysis
1. Authors must first test the normality of the data before comparative analyses.
2. Authors need to submit the data analysis details in Rmarkdown file to make the follow up of the data is clear and simple.

**Response**: We checked the data normality, it was much skewed, so we used the nonparametric test (Kruskal-Wasllis Test pairwise comparison and Mann-Whitney U Test) to analyse the data

Results
Line 166: It is not possible to start a sentence with numbers, please replace write “82.7%” in letters first

**Response**: Written in letter
Line 166: harmonize the writing of "park" in the manuscript; either "Park" or "park"

**Corrected:** park with small letter
Line 170-173: these results are specific to *Glossina pallidipes*, please specify, since the few *G. fuscipes* have been collected using sticky traps

**Response:** specified as recommended
Line 174: remove “(FTD)” in the table and replace the table tittle by “Mean density of tsetse flies (Fly per tap per day) in Nech Sar and Maze National Parks, southwest Ethiopia”

**Response:** corrected as recommended
Line 175: remove “FTD = Fly per trap per day”

**Response:** removed
Line 183: add standard deviations to histograms (Figure 2)

**Response:** Done
Line 185: remove “s” to “traps”

**Response:** Removed
Line 188: add “s” to “type” after vegetation

**Response**: added
Line 201: add “except in riverine forest” after “vegetation types…”

**Response**: added
Lines 208-210: Table 3 presents results of multiple comparisons, which was not specified in the "data analysis" section

**Response**: Kruskal-Wasllis Test pairwise comparison was done and corrected in the revised version

**Response**: Specified in the statistical analysis
Tables 3 and 4:
1.      for the column “Mean ± SE”, harmonize the number of digits after the decimal point

**Response**: Corrected
2.      what is the unit of “Mean ± SE”??

Response: corrected

Discussion
Delete from line 219 to line 224 because it is a repetition of the results

**Response**: deleted

Line 244: change “0” by “°”    “…200C and 340C….”

**Response**: Corrected

References
Write species names in italics

Do you want your identity to be public for this peer review?

Reviewer #1: Yes: Dr. Paul Mireji

Reviewer #2: No

Reviewer #3: No
