# Supplementary material for: Evaluating the efficacy of various traps in catching tsetse flies at Nech Sar and Maze National Parks, Southwestern Ethiopia: An Implication for Trypanosoma Vector Control
Source: PLoS Negl Trop Dis. 2022 Dec 22;16(12):e0010999. doi: 10.1371/journal.pntd.0010999 (PMC9822101; doi:10.1371/journal.pntd.0010999)
Supplement: Attachment — Submitted filename: Response.docx [file pntd.0010999.s003.docx]

In their manuscript, Asfaw et al. evaluate the efficacy of the NGU, Biconical and Sticky traps against different species of tsetse in different vegetation types*.* The contents of manuscript are of importance for tsetse vector control in Ethiopia. However, the manuscript requires addressing the following issues before it can be published.

Introduction

Line 91: remove the word “big”. The authors should also consider giving a summary of the tsetse eradication program stating the control and monitoring methods being used.

**Response**: Thank you for the comments. We removed the word big, and added the summary of control method

Line 98: sentence starting with “It will….” should be in past tense.

**Response**: We considered this

Materials and methods

Line 102: consider rewriting the first sentence to “Permission to conduct the study in the National Parks was obtained from xxxxx (Park Authorities in Ethiopia).

**Response**: Corrected

Lines 127 to 137: the authors should clearly indicate the number of Latin Square replicates per month per site in each vegetation. This could be one of the limitations of the study and should be highlighted in the discussion. The authors should also describe the traps in more detail. For example what were dimensions of the stick trap and what material was used to make the trap stick?

**Response**: Thank you for important question, which help us to explain more about the design. The number of Latin square replicates in each vegetation type and the total number of traps were explained in the text. Our calculation was based on the number of replicates indicated. We included the details about the traps

Lines 138 and 143: the authors should consider combining the subheadings “Tsetse fly collection” and “Tsetse fly species identification” into one subheading “Tsetse collection and species identification”.

**Response**: Done as recommended

Line 139: should be “baited with urine”. What was the source of the urine? Was the urine fresh? How was the urine dispensed?

**Response**: We explained in the text

Lines 155 to 162: the authors should indicate how the calculated the means (lines 176 to 177) and fly densities (185 to 186) reported in the results section.

**Response**: Included in the text

Results

Lines 166 to 170: the authors should consider indicating the actual numbers of each tsetse species collected by trap.

**Response**: we indicated the number of tsetse fly species

Line 169: write 82.7% in words

**Response**: We did it

Discussion

The authors should discuss the limitation of the study.

**Response**: We included in the last part of the discussion
